# The Clash of the Titans: COVID-19, Carbapenem-Resistant *Enterobacterales,* and First *mcr-1*-Mediated Colistin Resistance in Humans in Romania

**DOI:** 10.3390/antibiotics12020324

**Published:** 2023-02-03

**Authors:** Ionela-Larisa Miftode, Daniela Leca, Radu-Stefan Miftode, Florin Roşu, Claudia Plesca, Isabela Loghin, Amalia Stefana Timpau, Ivona Mitu, Irina Mititiuc, Olivia Dorneanu, Egidia Miftode

**Affiliations:** 1 Department of Internal Medicine II, Faculty of Medicine, University of Medicine and Pharmacy Gr. T. Popa, 700115 Iasi, Romania; 2 St. Parascheva Clinical Hospital of Infectious Diseases, 700116 Iasi, Romania; 3 Department of Internal Medicine I, Faculty of Medicine, University of Medicine and Pharmacy Gr. T. Popa, 700115 Iasi, Romania; 4 Department of Intensive Care Unit, Infectious Diseases Clinical Hospital, 700115 Iasi, Romania; 5 Department of Morpho-Functional Sciences II, Faculty of Medicine, University of Medicine and Pharmacy Gr. T. Popa, 700115 Iasi, Romania; 6 Department of Preventive Medicine and Interdisciplinarity, Faculty of Medicine, University of Medicine and Pharmacy Gr. T. Popa, 700115 Iasi, Romania

**Keywords:** carbapenem-resistant *Enterobacterales*, COVID-19, urinary tract infections, antibiotic resistance

## Abstract

(1) Background: Antibiotic resistance and coronavirus disease-19 (COVID-19) represent a dual challenge in daily clinical practice, inducing a high burden on public health systems. Hence, we aimed to dynamically evaluate the impact of COVID-19 on patients with carbapenem-resistant *Enterobacterales* (CRE) urinary tract infections (UTIs), as well as the antibiotic resistance trends after the onset of the pandemic. (2) Methods: We conducted a prospective study including patients with CRE UTIs who were enrolled both pre- and during the pandemic from 2019 to 2022. We further performed a standardized and comparative clinical, paraclinical, and microbiological assessment between patients with and without COVID-19. (3) Results: A total of 87 patients with CRE UTIs were included in this study (46 pre-pandemic and 41 during the pandemic, of which 21 had associated Severe Acute Respiratory Syndrome Coronavirus-2 infection). *Klebsiella pneumoniae* was the main etiological agent of the UTIs, with the majority of strains (82.7%) being carbapenemase producers (mainly OXA-48 producers), while five of the 34 colistin-resistant isolates were harboring the mobile colistin resistance-1 (*mcr-1*) gene. COVID-19 patients presented a significantly worse outcome with higher rates of intensive care unit (ICU) admissions (66.7% for COVID patients vs. 18.2% for non-COVID patients, *p* < 0.001), while the fatality rates were also considerably higher among patients with concomitant viral infection (33.3% vs. 12.1%, *p* < 0.001). Besides COVID-19, additional risk factors associated with increased mortality were urinary catheterization, sepsis with *K. pneumoniae*, impaired liver and kidney function, and an inappropriate initial empiric antibiotic therapy. (4) Conclusions: COVID-19 showed a pronounced negative impact on patients with CRE UTIs, with significantly longer hospitalizations and higher ICU admissions and mortality rates.

## 1. Introduction

Almost three challenging years have passed since Severe Acute Respiratory Syndrome Coronavirus-2 (SARS-CoV-2), a single-stranded RNA virus with positive polarity, emerged as the etiologic agent of Coronavirus disease-19 (COVID-19). Since then, COVID-19 has reached pandemic proportions, being responsible for more than 6.5 million deaths worldwide, especially among vulnerable patients with multiple associated pathologies [1]. The first COVID-19 case in Romania was reported on 26 February 2020, followed by a rapidly growing trend since then with significant peaks in incidence during successive pandemic waves, most prominently in October 2021.

The immunosuppression associated with SARS-CoV-2 infection predisposes to a variety of bacterial superinfections, thus significantly impacting the outcome of those patients [2]. To date, the involvement of superinfections or co-infections in aggravating COVID-19 has not been extensively studied [3]. In a large meta-analysis and systematic review, Lansbury et al. reiterated that 7% of the hospitalized COVID-19 patients had bacterial co-infections, with even higher rates in the Intensive Care Unit (ICU) settings [4,5]. There is a wide spectrum of incriminated pathogens reported so far, such as *Escherichia coli*, *Klebsiella pneumoniae*, *Pseudomonas aeruginosa*, *Haemophilus influenzae*, *Streptococcus pneumoniae*, *Staphylococcus aureus*, *Aspergillus fumigatus*, *Mycoplasma pneumoniae*, *Chlamydia pneumoniae*, *Legionella pneumophila*, and *Acinetobacter baumannii*, most of them causing respiratory tract infections [6,7,8,9].

A recently published Italian study reported that the incidence of MDR-associated bacterial infections was considerably higher in COVID-19 patients, compared to non-COVID-19 patients, with *K. pneumoniae* being the main etiological agent [10]. Similarly, as reported by Contou et al. in a monocentric French study, 28% of COVID-19 patients admitted in the ICU presented bacterial co-infections, while the principal etiological agent was *S. aureus*, followed by *H. influenzae* or *Enterobacterales* [5,11].

Other authors observed a much lower incidence of associated infections; for example, in the United Kingdom, out of a total of 836 patients, only 3.2% had an early co-infection, with *S. aureus* as the most commonly isolated pathogen [12].

Furthermore, worse outcomes have been noticed in hospitalized COVID-19 patients presenting with associated infections caused by multidrug-resistant (MDR) microorganisms. It is worth mentioning that strict measures and standardized protocols concerning infection control and prevention were implemented after the onset of the COVID-19 pandemic, determining a decreased occurrence of certain infections. However, in other situations, COVID-19 was associated with an increase in the incidence of bacterial, hospital-acquired infections [13,14].

Urinary tract infections (UTIs) caused by carbapenem-resistant *Enterobacterales* (CRE) represent a rising concern due to the paucity of therapeutical options available, especially in countries from Eastern Europe. Therefore, a dynamic assessment of the local isolates’ resistance patterns and constantly updated knowledge of the microorganisms most commonly involved are crucial in choosing an appropriate empiric antibiotic therapy for UTIs [15,16,17].

Therefore, we aimed to describe the burden and the impact of SARS-CoV-2-associated infections in patients with CRE UTIs, as well as the shift on antibiotic susceptibility of those isolates after the onset of the COVID-19 pandemic in the Eastern Europe.

## 2. Results

### 2.1. Baseline Characteristics

A total of 87 patients were included in this analysis. Of those, 46 were enrolled before the local onset of the COVID-19 pandemic. Of the 41 patients who were admitted during the pandemic, 21 presented a form of COVID-19.

The mean age of the cohort was 69.2 ± 12.3 years. The study population presented a symmetrical gender distribution, including 43 males (49.4%) and 44 females (50.6%), with a significant negative correlation between the presence of COVID-19 and male gender (r = −0.235, *p* = 0.028). Hospitalization length ranged between 1 and 81 days, with a mean value of 20 ± 17.7 days, significantly longer for COVID-19 patients (26.1 ± 15.7 days, r = 0.359, *p* = 0.001) (Table 1).

The most frequent causative agent of UTIs was *K. pneumoniae*, identified in 76 (87.3%) cases, followed by *Providencia stuartii* in 4 (4.5%) cases, *Enterobacter cloacae* in 3 (3.4%) patients, while *E. coli*, *Enterobacter aerogenes*, *Citrobacter freundii*, and *Serratia marcescens* accounted for one case (1.1%) each. Among the 21 COVID-19 patients, the UTIs were almost exclusively caused by *K. pneumoniae*, as we recorded only one isolated case in which *E. cloacae* was the etiological agent.

### 2.2. Resistance Patterns According to COVID-19

When analyzing the antibiotic resistance profiles before and after the onset of the COVID-19 pandemic, we found similar resistance rates, the only significant differences being in the case of gentamicin, with a decreased resistance, from 82.6% to 52.5% (*p* = 0.001), and in the case of ceftazidime + avibactam (CAZ–AVI), which presented a higher resistance, from 53.8% to 75.8% (*p* = 0.049). As one can observe in Table 2 and Table 3, the isolates were not entirely tested against all the antibiotics listed below.

### 2.3. Specific Resistance Profiles

**Figure 1:**
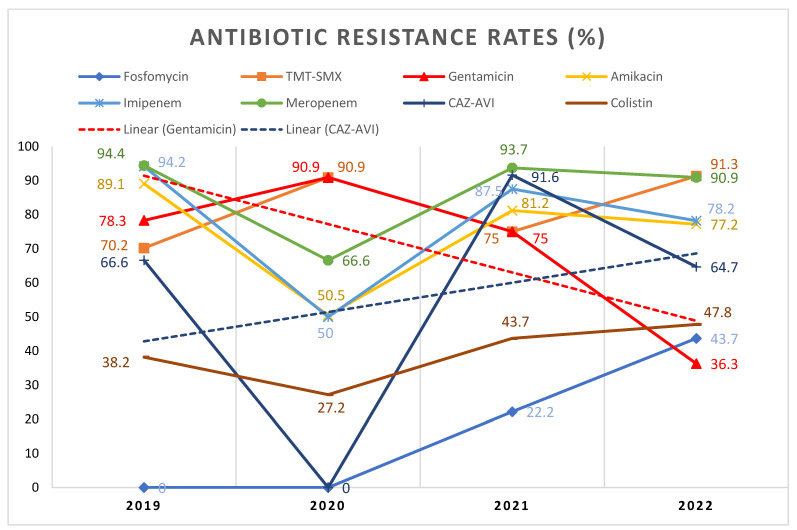
Antibiotic resistance rates during 2019–2022. The most significant changes (i.e., gentamicin and CAZ–AVI) are highlighted with supplementary linear trend lines. TMT-SMX—Trimethoprim-sulfamethoxazole; CAZ–AVI—Ceftazidime + avibactam.

Furthermore, while analyzing the resistance rates between 2019 and 2022, we noted that the incidence of resistant isolates has increased for most of the tested antibiotics, with the most significant decrease being observed for gentamicin, starting from a resistance of 78.3% in 2019 and then shrinking to only 36.3% in 2022, *p* = 0.0009 (Figure 1). It is worth mentioning that out of the 24 evaluated antibiotics, we identified a 100% resistance rate for seven (29.1%) molecules (amoxicillin + clavulanic acid, ampicillin + sulbactam, cefixime, cefuroxime, ceftazidime, cefotaxime, and ertapenem). Concerning the fluoroquinolones, the detected resistance was considerable, figuring above 96%, while for nitrofurantoin, only two isolates were susceptible (one identified in 2019 and one in 2022).

Of the 36 isolates resistant to CAZ–AVI, the majority were New Delhi metallo-β-lactamase (NDM)-producing microorganisms (13 isolates—36.1%), followed by oxacillinase (OXA)-48-type carbapenemases -producing microorganisms (nine isolates—25%), and *K. pneumoniae* carbapenemase (KPC) (five isolates—13.8%). In another five isolates, we did not identify any carbapenemases at all, while in three cases (8.3%), we detected two enzymes that were produced simultaneously. None of the patients had previous treatments that included CAZ–AVI.

**Figure 2:**
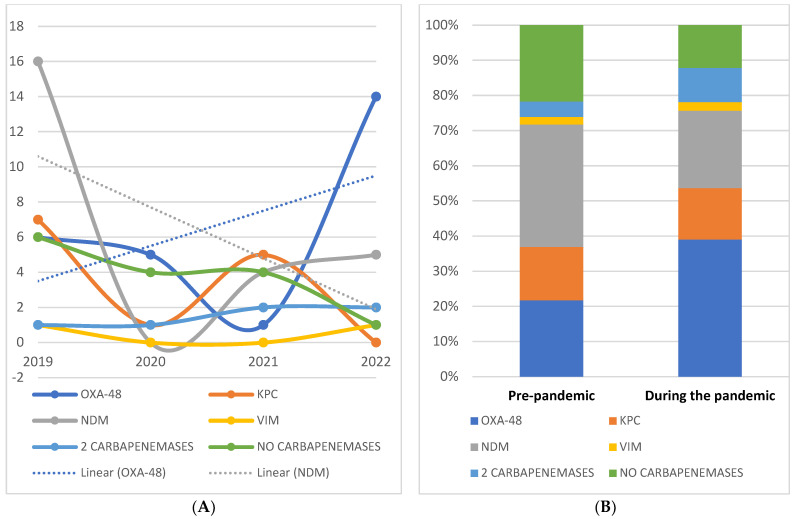
(**A**)—Chronological distribution of carbapenem-resistant isolates during 2019−2022. (**B**)—Types of carbapenemases production before and after the onset of the COVID−19 pandemic.

In order to identify the mechanism by which carbapenemase resistance occurred, we investigated the production of carbapenemases in each individual case (with a 100% concordance between the results obtained by phenotypic and genotypic methods). Thus, we observed that out of the 87 strains, 72 (82.7%) were carbapenemase-producing, as follows: 26 (36.1%) OXA-48-producing strains, 25 (34.7%) NDM-producing strains, 13 (18%) KPC-producing strains, two (2.7%) Verona integron-encoded metallo-β-lactamase (VIM)-producing strains, and in six (8.3%) cases, we observed the concomitant production of two carbapenemases, i.e., KPC + NDM in five cases and KPC + OXA-48 in one case. Additionally, we identified important chronological changes in the type of carbapenemase produced; starting from a predominance of NDM-producing isolates in 2019, representing 43.2% of cases, the trend shifted in 2022 towards OXA-48 as the main identified enzyme, observed in 60.8% of cases (Figure 2A).

While analyzing the strains isolated after the onset of the COVID-19 pandemic, we observed a decrease in both resistant, non-carbapenemase-producing isolates (10–21.7% pre-COVID vs. 5–12.2% during the pandemic, *p* = 0.128) and in NDM-producing isolates (16–34.7% vs. 9–21.9%, *p* = 0.09). However, the only statistically significant change was the increased prevalence of OXA-48-producing isolates (10–21.7% vs. 16–39%, *p* = 0.043) (Figure 2B).

In addition, for colistin-resistant strains, we performed the NG Test^®^ MCR-1 assay for identifying the mobile colistin resistance-1 (*mcr-1*) gene. Of the 34 isolates resistant to colistin, five (14.7%) were *mcr-1*-harboring. Three strains were isolated in 2019 and one strain in 2021 and 2022, respectively; in all cases, the isolated bacteria was *K. pneumonia*, without recorded fatalities and with a generally favorable outcome, as none of the patients required ICU admission.

In terms of therapeutical approach, most of the patients (65.5%) have received associations consisting of two or more antibiotics. The most common regimens include imipenem + cilastatin (28 patients—32.1%), colistin (27 patients—31%), meropenem (21 patients—24.1%), amikacin (20 patients—22.9%), trimethoprim-sulfamethoxazole (18 patients—20.6%), or gentamicin (17 patients—19.5%); other antibiotics, such as ciprofloxacin, nitrofurantoin, fosfomycin, or ertapenem were less used.

### 2.4. Risk Factors

Furthermore, we investigated the prognostic value of several relevant clinical and biological parameters for an unfavorable outcome. The presence of an indwelling urinary catheter, the associated bloodstream infections, or a concomitant respiratory pathology (either infectious or non-infectious), as well as impaired kidney or liver function, were significantly associated with increased mortality. Of note, the patients with a favorable outcome had a much shorter length of hospital stay (17.3 versus 32.9 days, *p* = 0.001) and were less likely to be transferred to the ICU (15.2% versus 100%, *p* < 0.0001) or to necessitate changes in the antibiotic therapy (47.2% versus 86.6%, *p* = 0.005) (Table 4).

Interestingly, although male gender and diabetes mellitus are generally identified as major risk factors for complicated UTIs and increased mortality, they were more prevalent among patients with a favorable outcome in our study.

### 2.5. Evolution, Prognosis, and Predictors of Poor Outcome

We identified a total of 15 (17.2%) fatalities, with no significant correlation between the type of carbapenemase produced and a negative outcome such as ICU transfer or death (Table 5).

When assessing the outcome of the patients, we identified a strong correlation between mortality and the coexistence of COVID-19 (r = 0.453, *p* < 0.0001); the subsequent logistic regression basically highlighted that a patient diagnosed with COVID-19 was 3.625 times more likely to die compared to a non-COVID control (CI: 1.125–11.68, *p* = 0.031) (Table 6).

Moreover, a concomitant diagnosis of COVID-19 was associated with an increased risk for ICU admission, which is per se an important predictor for negative outcome. Another classic and significant predictor of poor prognosis in patients with UTI was urinary catheterization.

**Figure 3:**
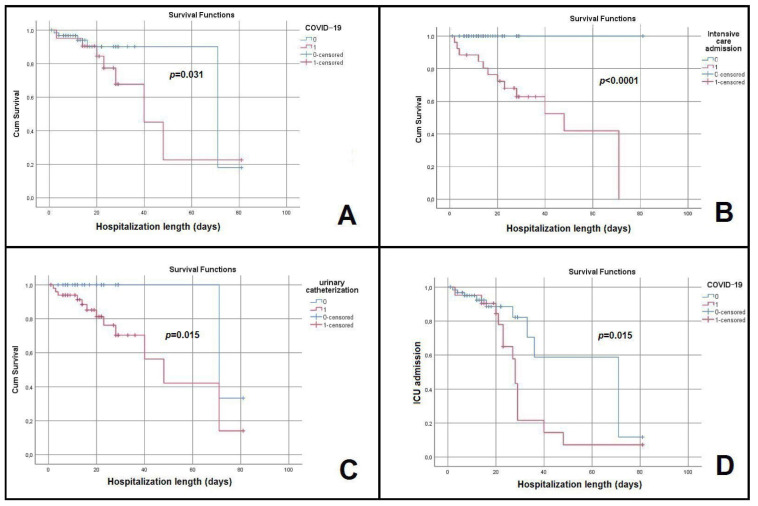
Cumulative proportions of mortality estimates (**A**)—between patients with (red line) and without (blue line) COVID−19; (**B**)—between patients admitted (red line) or not (blue line) in the ICU setting; (**C**)—between patients that were urinary catheterized (red line) or not (blue line). (**D**)—Cumulative proportions of ICU admission estimates between patients with (red line) and without (blue line) COVID−19.

The subsequent Kaplan-Meier survival curves are depicted in Figure 3.

## 3. Discussion

Viral respiratory infections, such as COVID-19 or even “classical” influenza, cause an impairment in both innate and adaptive antibacterial host defenses. Therefore, colonizing bacteria take advantage of this temporary vulnerability of the physical and immunological barriers, causing secondary infections which are usually associated with worse outcomes, particularly in frail patients with comorbidities [18,19,20,21]. However, most studies focused on the occurrence of respiratory tract infections, with only scarce data being reported on the association between UTIs and COVID-19.

In a systematic review, Musuuza et al. observed that 24% of COVID-19 patients have superinfections, while 19% have associated co-infections, both being significantly correlated with negative outcomes such as prolonged hospitalizations or increased mortality rates [22]. Moreover, Xu et al. reported that up to 72% of patients received antibiotic treatment [23] (including broad-spectrum regimens) during the therapeutic approach of critically ill COVID-19 patients, even without a complete ascertainment of a secondary bacterial infection [18]. Consequently, a decline in adhering to antibiotic stewardship programs during the pandemic has been observed, as healthcare providers focused primarily on the prompt and integrative antimicrobial treatment in COVID-19 patients [18,24].

The initial perception of high antibiotic consumption during the SARS-CoV-2 pandemic was influenced by early reports from Wuhan (China) indicating that up to 50% of patients who died from COVID-19 had a secondary bacterial infection [25,26]. Several studies also revealed a rather low prevalence of bacterial or fungal infections, but a persistently high use of broad-spectrum empirical antibiotics in COVID-19 patients [4,27,28]. Cumulatively, these factors may have a significant impact on the infection detection rates among COVID-19 patients, with increased suspicion of underreporting [29].

CRE are a group of microorganisms that cause some of the most difficult-to-treat infections; therefore, it is not surprising that as recently as 2013, the Centers for Disease Control and Prevention (CDC) has listed them as one of the three most urgent public health threats, with a rapidly spreading resistance worldwide [30]. Before the introduction of the association consisting of β-lactam + β-lactamase inhibitors active against carbapenemase-producing microorganisms, mortality rates for these infections ranged from 24% to as high as 74% [31,32]. Furthermore, knowledge of the type of carbapenemase produced may have implications for therapeutic management. For example, among older antibiotics, temocillin may be active against KPC-producing CRE [33], while ceftazidime or aztreonam usually retain activity against OXA-48-producing *Enterobacterales*, as long as the strains do not additionally produce other enzymes, such as AmpC or BLSE [34].

Moreover, the type of synthesized carbapenemase may predict the efficacy of new β-lactams + β-lactamase inhibitors associations. CAZ–AVI, meropenem + vaborbactam, and imipenem + relebactam are active in vitro on KPC-producing *Enterobacterales*. Moreover, in the case of the association of meropenem with vaborbactam and CAZ–AVI, some studies have demonstrated a superior potency compared to regimens including colistin, an antibiotic that is currently commonly used for the treatment of infections with carbapenem-resistant microorganisms, but which is equally associated with notable side effects [35,36].

CAZ–AVI regimen has additional activity over OXA-48-producing strains, since this enzyme has no activity on ceftazidime and avibactam inhibits BLSE, which may be simultaneously produced [37,38]. Thus, in our research, we identified that most of the isolates resistant to CAZ–AVI were NDM-producing strains, with significantly lower resistance rates to OXA-48- or KPC-producing isolates. However, the identification of KPC-producing microorganisms that are resistant to CAZ–AVI represents a major concern; a large systematic review from 2021, which included a total of 23 studies, identified only 57 isolates resistant to CAZ–AVI (none of them from Romania), of which only 19 showed baseline resistance (i.e., without previous exposure to a CAZ–AVI-based treatment) [39].

Consistent with the data reported in the literature [40,41,42,43,44], the most common etiological agent identified in our study was *K. pneumoniae*, responsible for 87.3% of cases, while the most commonly produced enzymes were OXA-48 and NDM, emphasizing the significantly increased incidence of OXA-48-producing isolates.

Mortality in CRE infections may be influenced by four categories of risk factors: those related to patient characteristics (such as age, gender, presence of comorbidities, or immunosuppression), the type and source of infection, the initial therapeutic approach (especially the delay in initiating appropriate therapy), and the characteristics of the microorganism (including the type of carbapenemase) [45,46]. Although we did not identify a significant influence on mortality depending on the type of produced carbapenemase, the association of COVID-19 had a strong correlation with mortality, which was almost three times higher compared to non-COVID patients. In addition, we identified a significant correlation (*p* < 0.0001) between a severe course of the disease, translated to the need for intensive support in an ICU setting, and COVID-19; this represents an additional risk factor for co-infections or superinfections with hospital-acquired, MDR microorganisms, especially if the patients are mechanically ventilated or have an indwelling urinary catheter [29,47].

In colistin-resistant strains, we searched for the presence of a plasmid-mediated resistance mechanism, i.e., the presence of the *mcr-1* gene, as it is the first colistin resistance gene to be identified [48] and currently the most common one [49]. We identified five positive cases. This finding represents a novelty at the national level given that, so far in Romania, no case of colistin-resistant strains through this mechanism has been identified in human medicine; in animals, this gene has been identified since 2011 in slaughterhouses from North-East region of Romania, with a prevalence of plasmid-mediated resistance of 11.9% [50]. Unlike most studies reporting *E. coli* as the main pathogen exhibiting this resistance mechanism [51,52], all of the strains exhibiting the *mcr-1* gene in our study were represented by *K. pneumoniae*.

Overall mortality for COVID-19 patients, although much higher than the one recorded among non-COVID-19-patients (33.3% versus 12.1%, *p* < 0.01), was still significantly lower than the 56.7% fatality rate reported by Vijay et al. for COVID-19 patients presenting associated infections. Of course, MDR infections were associated with increased risk of death, particularly those caused by *Enterobacterales* [53].

Dissemination of uropathogens via the hematogenous route had a significant impact on mortality, as virtually all five patients who had positive blood cultures died. Studies analyzing mortality in patients with sepsis due to carbapenem-resistant bacteria have also reported high mortality rates, ranging from 30% to 40% [32,54], up to 52% [55], or even 65% [56]. Additional markers of poor prognosis, such as the need for a prolonged hospitalization or for ICU admission, were significantly more prevalent among patients with COVID-19 and among those who ultimately died. It is worth mentioning that diabetes mellitus, a risk factor commonly related to increased mortality in both COVID-19 patients and in those with UTIs [57,58], was significantly associated with a favorable outcome (*p* = 0.016), even though it was more common among COVID-19 patients (47.6% versus 28.8% among non-COVID-19 patients). A possible explanation for this controversial result resides in the regular medical check-ups and increased health status awareness among diabetic patients, as compared to their non-diabetic counterparts, an aspect that is even more pronounced during the pandemic. In this regard, the increased attention concerning the implications of infectious diseases on public health burden, individual quality of life, and even on vital prognosis should be further leveraged to support the long-term antimicrobial resistance agenda, even if it is still too early to predict how COVID-19 will really affect antimicrobial resistance. Although it is not among the aims of our study, the economic aspect should not be neglected either, with the extra costs of broad-spectrum antibiotic therapy adding to the fixed costs associated with the pandemic (such as protection equipment, mechanical ventilation devices, additional medical staff), therefore inducing significant socio-economic burden, an effect particularly visible in less-developed regions, such as the Northeast Romania [59,60].

## 4. Materials and Methods

We designed a prospective study that was conducted in a high-addressability Romanian clinical hospital from the “St. Parascheva” Clinical Hospital of Infectious Diseases is a 300-bed university setting that includes both clinical and intensive care wards being the largest infectious diseases tertiary center from a densely populated region. The study was initiated before the occurrence of SARS-CoV-2, as we included patients admitted between 1 January 2019 and 30 June 2022, covering a period of time both before and during the pandemic.

### 4.1. Study Population

We consecutively enrolled all hospitalized patients who were diagnosed with a UTI caused by a CRE strain. Both community-acquired and hospital-acquired (with symptoms onset >48 h after admission) infections were accounted for.

Inclusion criteria: (i) all adult patients (≥18 years), (ii) with a clinical syndrome suggestive of UTI (such as dysuria, fever, urinary incontinence, frequency, suprapubic pain, hematuria, or pain in the lumbar region), (iii) pyuria [≥10 white blood cell count (WBC)/mm^3^], and (iv) the isolation of an *Enterobacterales* strain in urine culture [≥10^5^ colony forming units (CFU)/mL)] that presented resistance to at least one carbapenem. We included only one isolate per patient and excluded those with CRE colonization.

For antibiotic susceptibility testing, the disk diffusion method and the Microscan automated antibiogram were used. Isolates resistant to at least one carbapenem (according to the EUCAST criteria) were tested for carbapenemase production using the immunochromatographic test NG-Test^®^ CARBA5 (NG Biotech, Guipry, France), a rapid test able to simultaneously detect 5 types of carbapenemases: KPC, OXA, VIM, Imipenemases (IMP), or NDM. Numerous studies analyzed the sensitivity and specificity rates of this rapid test with very promising results; for example, Kanahashy et al. reported a sensitivity of 92.3%, a specificity of 100%, and a negative predictive value of 93.1% [61], with Liu et al. finding similar rates, with a 100% specificity and a 92.1% sensitivity rate [62]. Additionally, Huang et al. reported a noteworthy sensitivity of 100% and a specificity of 99% [63]. Moreover, for the first 60 isolated strains, we also performed a genotypic test using the Xpert^®^ CARBA-R test (Cepheid, Sunnyvale, CA, USA), a PCR test compatible with the GeneXpertR Dx System device, for the detection and differentiation of the *bla*_KPC_, *bla*_NDM_, *bla*_VIM_, *bla*_OXA-48_, *bla*_OXA-181_, and *bla*_IMP-1_ genes, which were associated with resistance to carbapenems.

In addition, for colistin-resistant strains, we used the NG-Test^®^ MCR-1 immunochromatographic assay (NG Biotech, Guipry, France) to identify the *mcr-1* gene. The research group led by Volland et al. developed and validated in 2019 the NG Test^®^ MCR-1 assay for the rapid detection of the *mcr-1* gene by using monoclonal antibodies obtained via the immunization of Biozzi mice in laboratories. Thus, they achieved 100% sensitivity and 98% specificity, and the test demonstrated the ability to detect even *mcr-2* genes at a cost of only a slightly lower specificity [64].

### 4.2. Statistical Analysis

A preliminary Kolmogorov-Smirnov test was performed to assess the normal distribution of parameters in the study population. The normally distributed variables were presented as means ± standard deviation while the not-normally distributed ones were reported as medians with interquartile range (IQR 25–75). Differences between different subgroups were assessed using an independent t-test or one-way ANOVA, as appropriate. Correlation analysis between two or more variables was performed using either Pearson’s rank (r) coefficients (for continuous variables) or Spearman’s r (for categorical variables).

To compare the mortality risk associated with certain resistance patterns, we used the log-rank test, while the Kaplan-Meier method was used to draw the survival curves.

A *p*-value of 0.05 was considered statistically significant in all the analyses. We used Microsoft Excel version 2013 software (Microsoft Corporation, Redmond, WA, USA) for initial data collection, while the statistical analysis was performed with SPSS version 23 (IBM, Armonk, VA, USA).

## 5. Conclusions

In the current investigation, we demonstrated that COVID-19 had a significant prognostic influence in patients with CRE UTIs. Moreover, we validated the importance of *K. pneumoniae* as a leading causal agent of CRE UTIs, both before and during the COVID-19 pandemic. The continuous improvement and implementation of prevention protocols and control measures are essential to reduce the spread of both SARS-CoV-2 and CRE, thus limiting the potentially severe, associated infections.

Whilst undoubtedly the current main focus of healthcare providers is controlling the COVID-19 pandemic, we should not lose sight of the longstanding threat represented by antibiotic resistance, especially if our current stewardship programs are disrupted during these unprecedented times. The global menace of antimicrobial resistance will most likely persist beyond the COVID-19 crisis; therefore, the updates from different geographical regions on the prevalence of infections caused by MDR microorganisms should be constantly gathered to determine its true impact.

## 6. Study Limitations

The number of the patients included in this study is rather limited, since local CRE prevalence is still relatively low. Moreover, after the onset of the COVID-19 pandemic, the addressability of the patients with UTIs significantly decreased in our region, as the medical contacts were limited to emergency presentations for several weeks.

## Figures and Tables

**Table 1:** Baseline characteristics of the study group.

	COVID-19 Patients (N = 21)	Non-COVID-19 Patients (N = 66)	*p*-Value	OR (CI 95%)
Mean age (years)	70 ± 12.2	68.9 ± 12.5	0.745	-
Male gender	6 (28.5%)	37 (56%)	0.02	0.31 (0.10–0.90)
Urinary catheterization	17 (80.9%)	34 (51.5%)	0.017	4.00 (1.21–13.16)
Previous hospitalizations	15 (71.4%)	47 (71.2%)	0.985	1.01 (0.34–2.99)
Previous antibiotic therapy	13 (61.9%)	33 (50%)	0.453	1.62 (0.59–4.43)
Rural area of residence	9 (42.9%)	31 (47%)	0.805	0.84 (0.31–2.27)
Inappropriate empirical therapy	19 (90.5%)	28 (43.1%)	<0.001	12.55 (2.69–58.41)
Associated pathologies				
Respiratory	19 (90.5%)	12 (18.2%)	<0.001	42.75 (8.75–208.71)
Cardio-vascular	14 (66.7%)	51(77.3%)	0.390	0.58 (0.20–1.72)
Neurological	9 (42.9%)	29 (43.9%)	0.998	0.95 (0.33–2.57)
Oncological	2 (9.5%)	20 (30.3%)	0.083	0.24 (0.05–1.13)
Digestive	11 (52.4%)	40 (60.6%)	0.613	0.71 (0.26- 1.91)
Diabetes mellitus	10 (47.6%)	19 (28.8%)	0.121	2.24 (0.82–6.16)
Chronic kidney disease	5 (23.8%)	22 (33.3%)	0.589	0.62 (0.202–1.92)
Hydronephrosis	0 (0%)	8 (12.1%)	0.094	0.73 (0.64–0.83)
rUTIs	1 (4.8%)	18 (27.3%)	0.034	0.13 (0.01–1.06)
Obesity	4 (19%)	8 (12.1%)	0.473	1.70 (0.45–6.36)
Chronic alcohol consumption	0 (0%)	14 (21.2%)	0.018	0.71 (0.61–0.82)
Smoking	0 (0%)	9 (13.6%)	0.106	0.73 (0.63–0.83)
Outcome				
ICU transfer	14 (66.7%)	12 (18.2%)	<0.001	9.00 (2.99–27.02)
Death	7 (33.3%)	8 (12.1%)	0.043	3.62 (1.12–11.68)
Hospitalization length	26.1 ± 15.7	18.1 ± 17.9	0.014	-

OR—odds ratio; CI 95%—95% confidence interval; rUTIs—recurrent urinary tract infections; ICU—intensive care unit.

**Table 2:** Antibiotic resistance rates: COVID-19 versus non-COVID-19.

Antibiotic	Non COVID-19 (N = 66)	COVID-19 (N = 21)	*p*-Value	χ^2^	OR (CI 95%)
Nitrofurantoin	8/10 (80%)	6/6 (100%)	0.187	1.37	UND
Fosfomycin	4/18 (22.2%)	5/10 (50%)	0.015	2.27	0.28 (0.05–1.50)
Trimethoprim–sulfamethoxazole	53/66 (80.3%)	16/21 (76.1%)	0.341	0.16	1.27 (0.39–4.11)
Cefepime	63/65 (96.9%)	21/21 (100%)	0.248	0.06	UND
Cefoxitine	31/33 (93.9%)	21/21 (100%)	0.184	1.32	UND
Gentamicin	50/65 (76.9%)	9/21 (42.8%)	0.002	8.55	4.44 (1.57–15.56)
Tobramycin	35/37 (94.5%)	21/21 (100%)	0.201	1.17	UND
Amikacin	51/65 (78.4%)	18/21 (85.7%)	0.250	0.52	0.60 (0.15–2.36)
Ciprofloxacin	65/66 (98.4%)	20/21 (95.2%)	0.241	0.74	3.25 (0.19–54.35)
Ertapenem	66/66 (100%)	21/21 (100%)	1	3.13	UND
Imipenem	53/63 (84.1%)	17/21 (80.9%)	0.363	0.11	1.24 (0.34–4.49)
Meropenem	56/63 (88.8%)	19/20 (95%)	0.238	0.65	0.42 (0.04–3.64)
Piperacillin + tazobactam	66/66 (100%)	20/21 (95.2%)	0.136	3.17	UND
Ceftazidime + avibactam	23/37 (62.1%)	13/18 (72.2%)	0.242	0.54	0.63 (0.18–2.15)
Colistin	22/63 (34.9%)	12/21 (57.1%)	0.041	3.22	0.40 (0.14–1.10)

χ^2^—chi square test; OR—odds ratio; CI 95%—95% confidence interval; UND—Undefined.

**Table 3:** Antibiotic resistance rates: before and after the onset of the COVID-19 pandemic.

Antibiotic	Pre-Pandemic (N = 46)	During the Pandemic (N = 41)	*p*-Value	χ^2^	OR (CI 95%)
Nitrofurantoin	0/1 (0%)	14/15 (93.3%)	0.062	7.46	UND
Fosfomycin	0/3 (0%)	9/25 (36%)	0.147	1.59	UND
Trimethoprim– sulfamethoxazole	35/46 (76%)	34/41 (82.9%)	0.224	0.61	0.65 (0.22–1.88)
Cefepime	46/46 (100%)	39/41 (95.1%)	0.109	2.29	UND
Cefoxitine	46/46 (100%)	31/33 (93.9%)	0.085	2.86	UND
Gentamicin	38/46 (82.6%)	21/40 (52.5%)	0.001	9.00	4.29 (1.60–11.48)
Tobramycin	46/46 (100%)	36/38 (94.7%)	0.100	2.48	UND
Amikacin	38/46 (82.6%)	31/40 (77.5%)	0.283	0.35	1.37 (0.47–3.99)
Ciprofloxacin	45/46 (97.8%)	40/41 (97.5%)	0.471	0.006	1.12 (0.06–18.58)
Ertapenem	46/46 (100%)	41/41 (100%)	0.115	2.18	UND
Imipenem	37/43 (86%)	33/41 (80.4%)	0.256	0.46	1.49 (0.46–4.75)
Meropenem	38/43 (88.3%)	37/40 (92.5%)	0.277	0.40	0.61 (0.13–2.76)
Piperacillin + tazobactam	46/46 (100%)	39/40 (97.5%)	0.232	1.16	UND
Ceftazidime + avibactam	14/26 (53.8%)	22/29 (75.8%)	0.049	2.93	0.37 (0.11–1.17)
Colistin	15/43 (34.8%)	19/41 (46.3%)	0.148	1.14	0.62 (0.25–1.49)

χ^2^—chi square test; OR—odds ratio; CI 95%—95% confidence interval; UND—Undefined.

**Table 4:** Additional risk factors associated with mortality.

Parameter	Deceased (N = 15)	Survivors (N = 72)	χ^2^	*p*-Value	RR	CI 95%
Mean age (years)	66.6 ± 12.075	69.7 ± 12.436	-	0.368	-	-
Male gender	6 (40%)	37 (51.3%)	0.64	0.422	0.77	0.40–1.50
Rural area of residence	8 (53.3%)	32 (44.4%)	0.39	0.529	1.20	0.69–2.05
Urinary catheterization	13 (86.7%)	38 (52.8%)	5.87	0.015	1.64	1.22–2.20
*K. pneumoniae* sepsis	5 (33.3%)	0	25.46	<0.0001	-	-
Associated respiratory pathologies	10 (66.6%)	21 (21.1%)	7.61	0.005	2.28	1.37–3.79
Diabetes mellitus	1 (6.6%)	28 (38.8%)	5.80	0.016	0.17	0.02–1.16
Previous hospitalization	11 (73.3%)	51 (70.83%)	0.03	0.845	1.03	0.73–1.45
Previous antibiotic treatment	8 (53.3%)	38 (52.7%)	0.001	0.968	1.01	0.60–1.70
CKD	4 (26.6%)	23 (31.9%)	0.16	0.687	0.83	0.33–2.06
rUTIs	5 (33.3%)	14 (19.4%)	1.40	0.236	1.71	0.72–4.03
Urea (mg/dL—mean value)	148.4 ± 118.006	68.9 ± 58.071	-	0.0001	-	-
Creatinine (mg/dL—mean value)	2.2 ± 2.164	1.4 ± 1.033	-	0.047	-	-
ASAT (UI/L—mean value)	1021.6 ± 2474.638	37.6 ± 57.283	-	0.0008	-	-
GGT (UI/L—mean value)	214.5 ± 227.158	82.5 ± 118.862	-	0.001	-	-
Length of hospital stay	32.9 ± 26.908	17.3 ± 13.908	-	0.001	-	-
ICU transfer	15 (100%)	11 (15.2%)	42.52	<0.0001	6.54	3.79–11.27
Inadequate empirical therapy	13 (86.6%)	34 (47.2%)	7.77	0.005	1.83	1.33–2.51

χ^2^—chi square test; RR—relative risk; CI 95%—95% confidence interval; CKD—chronic kidney disease; rUTIs—recurrent urinary tract infections; ASAT—Aspartate aminotransferase; GGT—gamma-glutamyl transferase; ICU—intensive care unit.

**Table 5:** Correlation between the type of carbapenemase produced/COVID-19 and the patients’ outcome.

	ICU Transfer			Death		
UTIs with an OXA-48-producing strain (N = 26)	11 (42.3%)	r	0.177	5 (19.2%)	r	0.034
		*p*	0.101		*p*	0.752
UTIs with a KPC-producing strain (N = 13)	5 (38.4%)	r	0.079	3 (23%)	r	0.065
		*p*	0.470		*p*	0.551
UTIs with a NDM-producing strain (N = 25)	5 (20%)	r	−0.137	4 (16%)	r	−0.021
		*p*	0.205		*p*	0.848
UTIs with two carbapenemases- producing strain (N = 6)	2 (33.3%)	r	0.021	2 (33.3%)	r	0.116
		*p*	0.850		*p*	0.285
UTIs with a non-carbapenemase-producing strain (N = 15)	3 (20%)	r	−0.099	1 (6.6%)	r	−0.128
		*p*	0.364		*p*	0.238
COVID-19 patients (N = 21)	14 (66.6%)	r	0.453	7 (33.3%)	r	0.240
		*p*	<0.0001		*p*	0.025

UTIs—urinary tract infections; ICU—intensive care unit.

**Table 6:** Binary logistic regression evaluating the relationship between COVID-19 and mortality.

Variables in the Equation								
	B	S.E.	Wald	df	Sig.	Exp(B)	95% C.I. for EXP(B)	
							Lower	Upper
COVID 19	1.288	0.597	4.652	1	0.031	3.625	1.125	11.683
a. Variable(s) entered: COVID 19.								

## Data Availability

Not applicable.
